# Transcriptomic Analysis of Soil Grown *T. aestivum* cv. Root to Reveal the Changes in Expression of Genes in Response to Multiple Nutrients Deficiency

**DOI:** 10.3389/fpls.2017.01025

**Published:** 2017-06-22

**Authors:** Saurabh Gupta, Brijesh S. Yadav, Utkarsh Raj, Shiri Freilich, Pritish K. Varadwaj

**Affiliations:** ^1^Department of Bioinformatics, Indian Institute of Information TechnologyAllahabad, Allahabad, India; ^2^Department of Molecular Biology and Ecology of Plants, Tel Aviv UniversityTel Aviv, Israel; ^3^Institute of Plant Sciences, Newe Ya'ar Research Center, Agricultural Research OrganizationRamat Yishay, Israel

**Keywords:** nutrient deficiency, wheat root, stress, meta-analysis, metabolic pathways, gene ontology

## Abstract

Deficiency of necessary macronutrients, i.e., Potassium (K), Magnesium (Mg), Nitrogen (N), Phosphorus (P), and Sulfate (S) in the soil leads to a reduction in plant growth and yield, which is a result of changes in expression level of various genes. This study was performed to identify the differentially expressed genes and its associated metabolic pathways occurred in soil grown wheat root samples excavated from the control and treated fields. To identify the difference in gene expression levels due to deficiency of the said nutrients, a transcriptomic, meta-analysis was performed on array expression profile data. A set of 435 statistically significant probes encoding 398 Nutrient Deficiency Response Genes (NRGs) responding at-least one nutrients deficiency (ND) were identified. Out of them 55 NRGs were found to response to minimum two ND. Singular Enrichment Analysis (SEA) predicts ontological based classifications and functional analysis of NRGs in different cellular/molecular pathways involved in root development and growth. Functional annotation and reaction mechanism of differentially expressed genes, proteins/enzymes in the different metabolic pathway through MapMan analysis were explored. Further the meta-analysis was performed to revels the active involvement each NRGs in distinct tissues and their comparative potential expression analysis in different stress conditions. The study results in exploring the role of major acting candidate genes such as Non-specific serine/threonine protein kinase, Xyloglucan endotransglucosylase/hydrolase, Peroxides, Glycerophosphoryl diester phosphodiesterase, S-adenosylmethionine decarboxylase proenzyme, Dehydrin family proteins, Transcription factors, Membrane Proteins, Metal binding proteins, Photosystem proteins, Transporter and Transferase associated in different metabolic pathways. Finally, the differences of transcriptional responses in the soil-grown root of *T. aestivum* cv. and *in-vitro* grown model plants under nutrients deficiency were summarized.

## Introduction

Potassium (**K**), Magnesium (**Mg**), Nitrogen (**N**), Phosphorus (**P**), and Sulfate (**S**) are necessary macronutrients present in the soil for plant growth and development. Each nutrient acts in a specific manner in a variety of physiological and morphological aspects of plant growth. The plant growth and development is a result of different cellular and metabolic processes (Marschner and Rimmington, 1988; Maathuis, 2009). The **K** is an essential micronutrient which takes up about 2–10% of total dry weight in plants (Leigh and Wyn Jones, 1984). Earlier study reported that the **K** plays an important role in enzyme activation, protein synthesis, photosynthesis, turgor pressure, osmoregulation, and electrical neutralization (Römheld and Kirkby, 2010). Hence, the deficiency of the **K** brings many negative impacts on plant growth due to inappropriate osmotic pressure, nutrition imbalance and changes in photosynthesis, protein synthesis and other molecular effects. The deficiency of the **Mg** leads to improper functioning of chlorophyll, required for capturing the sun's energy during photosynthesis process (Cakmak and Yazici, 2010). The cellular molecules such as adenosine triphosphate (ATP), amino acids, nucleic acids, chlorophyll, phospholipids and other plant hormones are made up of **N, P**, and **S** (Takehisa et al., 2013). Among these nutrients, the larger quantity of the **N** is required for the production of said bio-molecules therefore it leaches faster from the soil. It is essential in the development of chlorophyll and plays an important role in the stimulus of photosynthesis. The **P** is crucial macronutrient at the early stage of plant growth and development as it is required for maintaining the various processes within the plant. Similarly, the **S** also plays an important role in plant growth because of its involvement in the production of various metabolites such as amino acids, proteins, sulpho-lipids and production of chlorophyll (Sorin et al., 2015).

Wheat (*T. aestivum*) is a cereal grain crop cultivated worldwide and became second largest cereal crop in terms of yield after maize in 2016 (Crosson and Frederick, 2016). Recently, yield losses were observed in this crop due to different abiotic and biotic stress. Under abiotic stress, the ND affect majorly in the quality and quantity of the crop. Identification and understanding of the cellular mechanism of signaling, sensing and adaptation in response to ND is crucial to enhance productivity and quality of the wheat (Gill et al., 2016; Gupta et al., 2016b, 2017). Considering the complexity of above said mechanisms and processes, it is crucial to understand the functional elements and molecular constituents involved during these NDs at transcriptome level. The transcriptomic analysis provides genome-wide measurement of messenger RNA (mRNA) expression levels based on microarray technology. Microarray technology is rigorously used to uncover the regulatory, signaling, sensing and adaptation mechanism of transcripts in response to nutrients deficiency (mainly **N, P**, and **K**) in model plants (Wasaki et al., 2003, 2006; Wu et al., 2003; Armengaud et al., 2004; Misson et al., 2005; Lian et al., 2006; Li et al., 2010a,b; Krapp et al., 2011; Cai et al., 2012; Ma et al., 2012; Park et al., 2012). These *in-vitro* grown customized experiments elucidate that several transcriptional responses occur when plants were subjected to various nutrients deficiency. Gruen et al. (2015) performed gene expression profiles of the soil-grown wheat root to analyze the effect of combined nutrient deficiencies (https://www.ncbi.nlm.nih.gov/geo/query/acc.cgi?acc=GSE61679). They have generated transcriptomic data of wheat root samples grown in limited nutrients field known as soil grown, which is different from *in-vitro* grown samples. The major difference in the said soil grown experiment with other *in-vitro* grown samples are (i) difference in types of soil, (ii) availability of inadequate nutrients in soils, and (iii) variation in environmental conditions. Hence the soil grown experiment conducted by Gruen et al. is found to be more close to the actual cultivation field conditions. This inspires us to carry a differential expression study on said soil grown data and to compare and verify its results with other *in-vitro* grown samples. In the present study, nutrient deficiency responsive genes (NRGs) in response to distinct NDs conditions were explored. Along with GO term, MapMan based analysis of each NRGs helps in the identification of function and their role in metabolic pathways. The meta-analysis was performed to uncover the NRGs involvement in distinct tissues of wheat and expression potential in different ND and stress conditions using 109 wheat root specific samples (Hughey and Butte, 2015). Used wheat root specific samples were retrieved from NCBI-GEO and EBI-ArrayExpress databases. This meta-analysis gives insights into the features of NRGs, which play crucial role in biological systems of wheat plant under abiotic stress. Finally, this study fills the gaps of earlier transcriptomic studies performed on model plants and soil-grown wheat, with an aim to identify the genes responsible in nutrient uptake mechanism.

## Materials and methods

### Microarray data and experiment

Microarray data, (Accession no. GSE61679) was downloaded in order to identify the transcriptional responses in the root of wheat, grown in soil with limited nutrients availability. To generate transcriptomic data, the root material was excavated in triplicate at booting stage (At this stage, development of head within the sheath of the flag leaf becomes visible) from the control fields with all nutrients supply known as control sample and treated samples with limited nutrient supply in the fields. These results of 6 treatments (one control and five treated samples) for each replicate, altogether 18 samples of root material were collected for all the 3 replicates. The root growing environmental conditions fluctuates as per day/night weather and the average temperature reported was 18°C (day)/10°C (night). Further, the total RNA was extracted using modified protocols of Verwoerd et al. (1989) with an additional phenol-chloroform-isoamylalcohol extraction of the aqueous phase after the first centrifugation and subsequent treatment with RNAse-free DNAse [Promega, Madison, USA]. In addition, silica-membrane purification kit was used to purify the total extracted RNA [RNeasy Plant mini kit, Qiagen, Maryland, USA]. Further, the biotin labeling of cDNAs was performed and the biotin-modified cRNAs were obtained from 500 ng total RNA using the Affymetrix 3′ IVT Express kit [Affymetrix User Manual P/N 702646 rev.7]. The hybridization performed in GeneChip® Hybridization, Wash, and Stain Kit was used for cRNA hybridization according to the user manual P/N 702731 of GeneChip®Expression wash. After hybridization of the gene chips were washed in the Affymetrix GeneChip fluidics station 450 [Fluidics script EukGE-WS2v5]. Gene chip scanning was performed using the Affymetrix®GeneChip®Scanner 3000, with default settings of Affymetrix®GeneChip®Command Console®Software(AGCC). The whole experiment was executed in Affymetrix®GeneChip®Wheat Genome array platform (Accession no. GPL3802). This array contains 61,127 probe sets representing 55,052 transcripts for all 42 chromosomes of the wheat genome (http://www.affymetrix.com).

### Microarray data analysis

The images of probe set signal values were examined through Affymetrix®GCOS 1.0 (MAS 5.0) software to generate summarized raw signal values in .CEL files. The raw data contains three biological replicates for each nutrient deficiency, including a control sample. A candidate gene was considered as expressed, when it appears in all these three replicates after RMA normalization, which is an inbuilt algorithm in GeneSpring GX 13.0 [Agilent Technologies, Inc]. For Affymetrix gene expression data, GeneSpring tool has two basic workflows i.e., (i) Analysis: Biological significance and (ii) Data import wizard. In our analysis, we have used 6 basic steps i.e., (1) Summary Report, (2) Experiment Grouping, (3) Quality check (QC) check on samples, (4) Filter Probe Sets, (5) Statistical Analysis, and (6) Fold Change of Analysis: Biological Significance workflow. Initially the summary report and grouping (Control vs. Nutrients deficient) for all sample replicates were generated. The QC of data was carried out using the Boxplot, Hierarchical clustering and Principal Component Analysis (PCA) (Raychaudhuri et al., 2000). The PCA calculates the principal components and contribution of an observation to component which is also known as contribution ratio, can be obtained by the ratio of the squared factor score of the observation by the eigenvalue associated with that component. Formally, the contribution of observation i to component j is, known as *Ctr*_*i, j*_ and obtained by: ${Ctr}_{i,j}=f_{i,j}^{2}/\delta_{j}$ equation, where δ_*j*_ eigenvalue of jth component and $f_{i,j}^{2}$ is factor score for the principal components (Abdi and Williams, 2010). The boxplot shows the shape of the data distribution along with its central value (median) and variability (inter-quartile range). The hierarchical clustering is used to look the patterns of gene expression values in array data and PCA computes all possible principal components and shows it in a 3D scatter plot. The quantitative analysis (QA) was carried out through an initial filtering using percentile shift method with default parameter values. Thereafter in subsequent steps, the statistical and fold change analysis was carried out on filtered probe sets. A probe/gene was considered as differentially expressed OR denoted as NRGs, when it satisfied (i) Fold change (FC) value FC value = +2 for up-regulated or FC value = −2 for down-regulated genes (calculated for each gene of ND samples based on control sample) and (ii) corrected *P*-value = 0.05 (*P*-value computation was carried out through *t*-test utilizing Benjamini-Hochberg FDR as a multiple testing correction methods). Identification significant probe sets encoded genes IDs, name and their description were manually extracted from Genome array file (Accession no: GPL3802) and also revalidated using PLEXdb (http://www.plexdb.org/) and ARTRAdb (http://artra.kazusa.or.jp/artra/ARI3_101/index.html) databases.

### Evaluation of gene ontological relation of NRGs

To understand various biological processes, molecular mechanism resulted due to the different ND in the wheat root, SEA of NRGs were performed through AgriGO analysis toolkit (http://bioinfo.cau.edu.cn/agriGO/). SEA analysis was performed using Fisher Test with 0.05 level of significance and Benjamini-Hochberg-Yekutieli Multi-test adjustment method using *T. aestivum* genome as a reference genome (Du et al., 2010; Gupta et al., 2016a).

### MapMan based analysis

MapMan analysis used to find the role of specific gene in a metabolic pathway using prior MapMan annotations scheme (http://mapman.gabipd.org/web/guest/mapmanstore) and in-house PERL programs as in Yadav et al. (2016) and Thimm et al. (2004). The macronutrients controlling genes were classified into a functional category within the hierarchy of MapMan pathway scheme (also known as BINCODE and file was downloaded from MapMan recourse). Further, the pathway enrichment analysis of functional category was performed by calculating the cumulative hypergeometric *P*-value. It is a probability of a gene group over-represented within a functional BINCODE at a rate higher than chance expectation. The calculation of the hypergeometric *P*-value for 398 differentially expressed genes over 55052 sample genes in 9 different situations (i.e., Up-regulated genes under K, N, P, Mg, and S deficiency and Down-regulated genes under K, N, P, and Mg deficiency) was carried out in which 4182 genes are found to be associated with different metabolic pathways. Several tests were executed for all sets of functional gene categories at different hierarchical levels. The significant hypergeometric *P*-value of a gene/a genes group was used for generation of heatmap in the R.

### Investigation and meta-analysis of NRGs

Gene investigation for all NRGs through Java based tool Genevestigator was carried out. This tool contains 2101 samples of different array experiments performed for wild type and non-wild type wheat in response to different stress conditions in its database [Genevestigator, Inc]. These wheat specific microarray experiments samples were fetched from NCBI-GEO and EBI-Array express. Since our analysis belongs to wheat root, exposed under different nutrients deficiency and generated through Affymetrix®GeneChip®wheat genome Array platform. Therefore, only the root specific 109 samples were found to be selected for this analysis. Genevestigator tool platform provides two types of analysis (i.e., Single Experiment Analysis and Compendium Wide Analysis). The Single Experiment Analysis is used to visualize the expression of NRGs across individual experiments and Compendium Wide Analysis is used to easily visualize the expression across thousands of experiments in a single analysis (Hruz et al., 2008). In both analysis, NRGs were used as input genes to perform meta-analysis while comparing with selected samples. Functional annotation of characterized/uncharacterized proteins was performed using standard protocol used by Gupta et al. (2016b).

## Results

### Identification of nutrient deficient response genes

**Figure 1:**
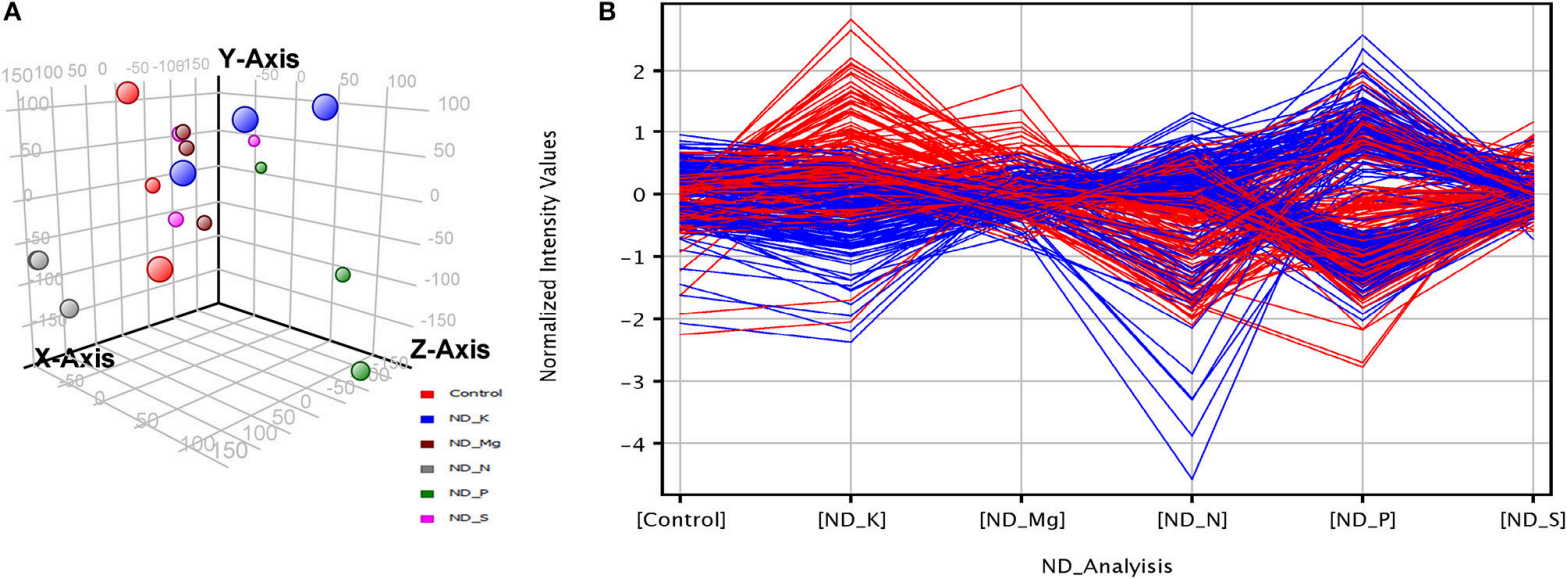
Overall signature of transcriptome profiles in wheat root in response to K, P, N, Mg, and S deficiency. **(A)** 3D scatter plot shows a score for each sample (scaled as X, Y, Z axis point) after applying Principal component analysis on 18 normalized data (3 replicated for 5 ND and 1 as Control). **(B)** Baseline transformation plot shows normalized intensity values of differentially expressed NRGs (calculated after rescaling the gene intensity to the same relative abundance level centering to zero). Red and blue line indicates two different expression profiles and each having similar genes.

**Figure 2:**
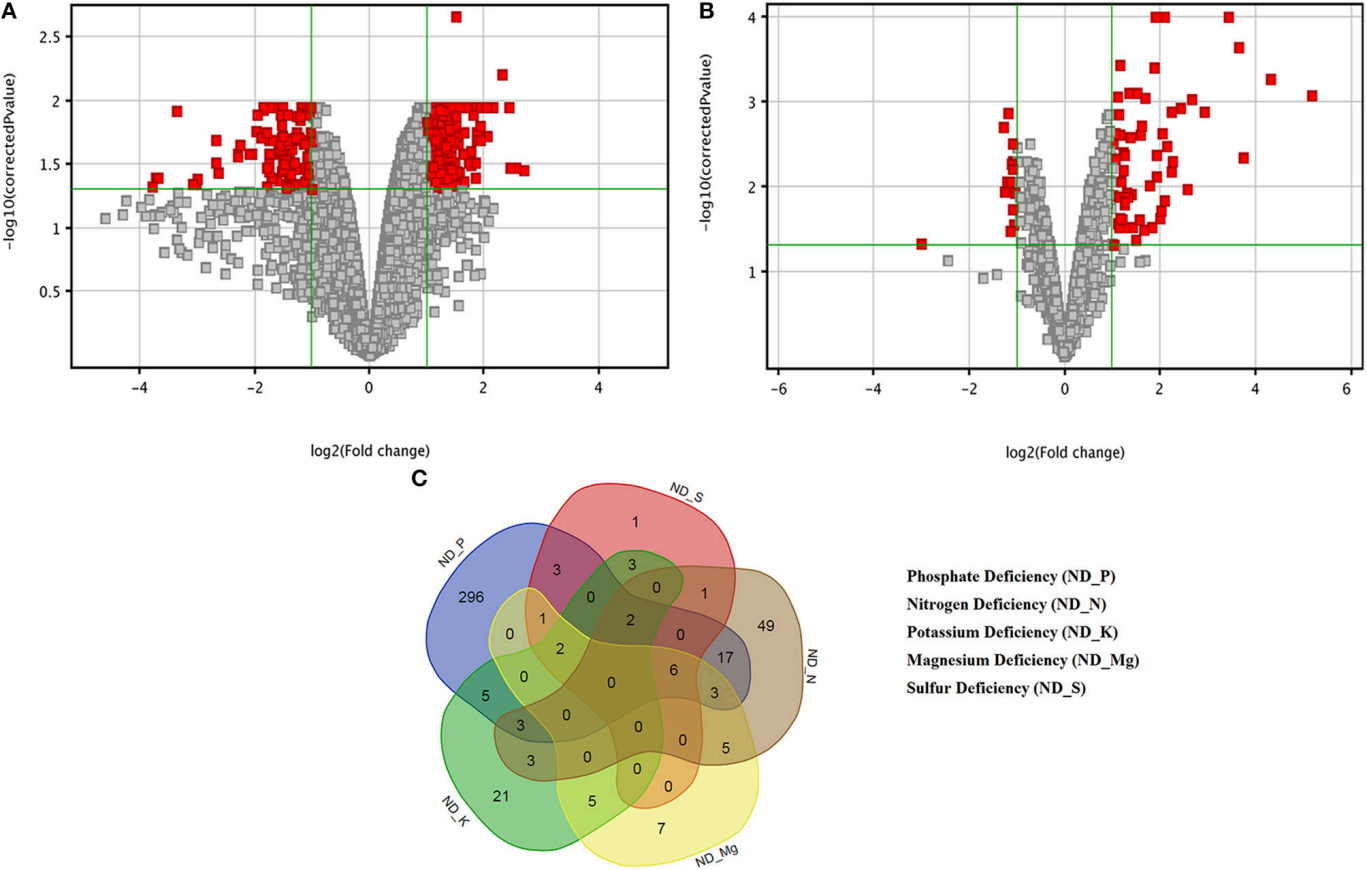
Volcano plots and Venn diagram of differentially expressed genes (DEGs) in response to nutrients deficiency. Volcano plots under **(A)** Phosphate deficiency (ND_P) and **(B)** Nitrogen deficiency indicating the DEGs in Red boxes. **(C)** Five-set Venn diagram of commonly expressed and unique DEGs for a particular nutrients deficiency.

**Figure 3:**
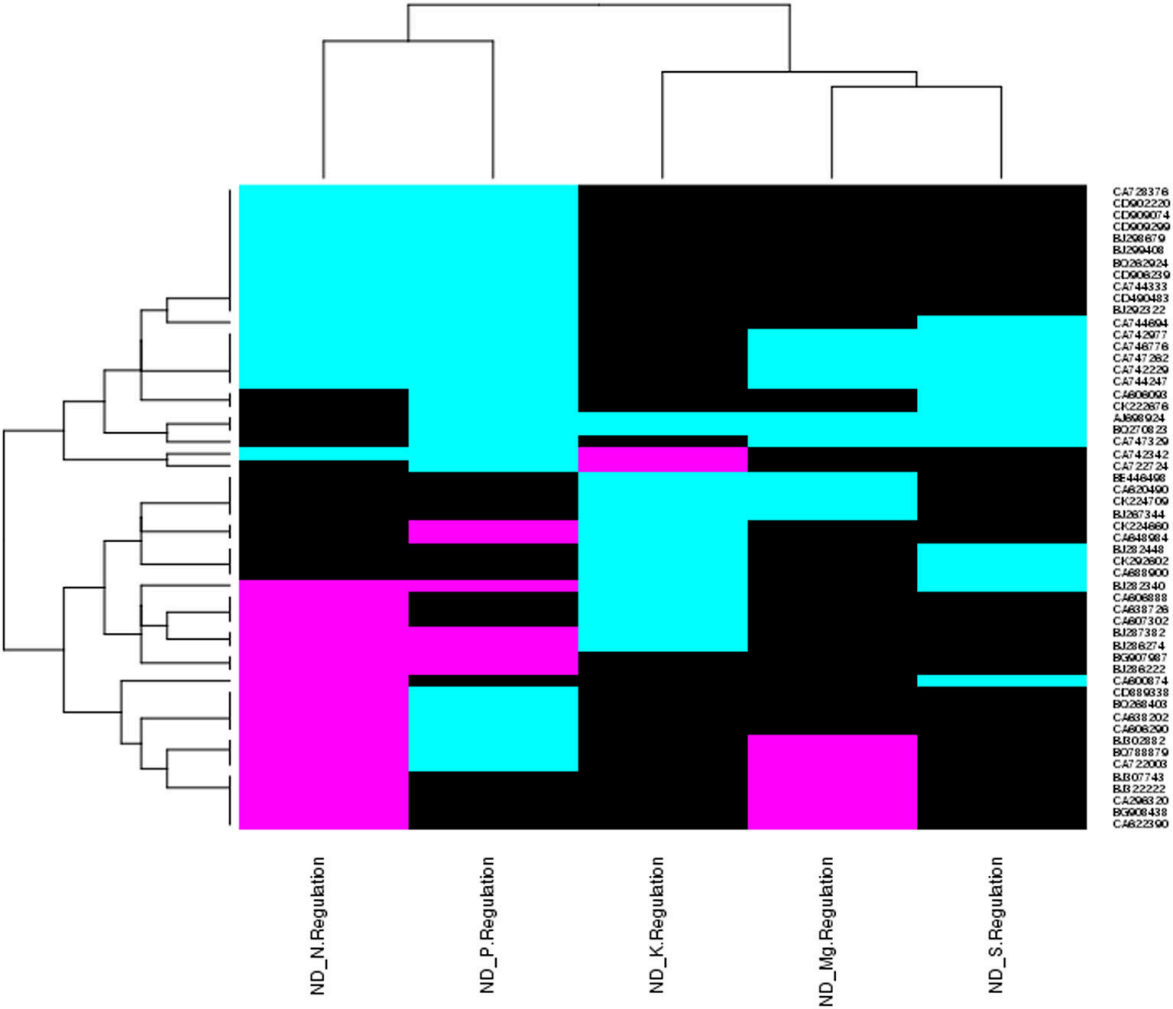
Heat map diagram shows genes expression patterns of 55 NRGs across multiple nutrients deficiency. Each column represents a single nutrient deficiency and each row represents a single transcript. Expression levels are colored green for up-regulated, red for down-regulated and black for no expression were found with respect to particular nutrient deficiency. At the left of the diagram the row wise gene ids and at bottom of the diagram name of the treatments are depicted.

The triplicate samples of roots of wheat were excavated at booting stage from the control soil as well as **K, Mg, N, P**, and **S** nutrients limited plots and total RNA was extracted by Guren et al. (2015). Further, Affymetrix®GeneChip®wheat genome Array platform generates total 18 raw signal values file (.CEL files) and used in GeneSpring tool for biological significance analysis (Bolstad et al., 2003). Normalization and other basic steps of biological significance analysis as discussed in methodology section were executed. Boxplot indicates shape of data distributions across all the samples after normalization (Figure S1). The dendrogram resulted from hierarchical clustering indicates the similar expression patterns of microarray data (Figure S2). The PCA was performed with an aim to simplify the description and analyze the structure of the samples of the data sets. In this analysis, the contribution ratio was also calculated for all treated samples with respect to control sample component. The projection of each sample on the principal component is represented through a 3D scatter plot (Figure 1A). It indicates one point for each array and color them accordingly by grouping as per experiment factors through which one can visualize the separations between groups of replicates. In this analysis, each sample replicate falls within a group or clustered together, while getting separated from other sample groups (Raychaudhuri et al., 2000). The **Mg** deficiency group replicates cluster is found to have similar pattern as in the control replicates cluster (Figure 1A). The QA of probes using percentile shift method resulted in 52,083 probes qualifying 0.75 quintile threshold value. Normalized probe sets were statistically tested using *t*-test (FDR = 0.05) and fold change analysis (FC ≥ +2.0 or FC ≤ −2.0) to check whether the candidate is differentially expressed in at least 1 out of 5 ND conditions against the control sample. This analysis identified NRGs, which qualify the defined screening threshold (Figure 1B) and their corresponding FC values are listed in Table S1. Analysis imitates 42 up-regulated and 3 down-regulated probes under limited **K** (ND_K); 21 up-regulated and 8 down-regulated probes under limited **Mg** (ND_Mg); 23 up-regulated and 66 down-regulated probes under limited **N** (ND_N); 101 up-regulated and 237 down-regulated probes under limited **P** (ND_P) and only 19 up-regulated genes under limited **S** (ND_S) were identified. Large numbers of NRGs under ND_P condition (Figure 2A) in root were expressed while comparing with other ND. Higher number gene expression lead to important changes in metabolic process such as alteration in lipid metabolism, compound rearrangement in cell wall, carbon supply acceleration for organic acid synthesis as earlier reported in rice root (Wasaki et al., 2003; Li et al., 2010b). The second highest numbers of genes were expressed under ND_N (Figure 2B). These genes are associated with diverse cellular function i.e., organ development, protein biosynthesis etc. (Gruber et al., 2013). Venn diagram illustrating the number of common genes and unique genes expressed among **N P K Mg**, and **S** deficiency and their sub sets are shown in Figure 2C. Out of 435, total 58 probes representing 55 transcripts were found to be responsible for at least two NDs, which indicates their importance in comparison to others genes (Table S2). The expression patterns of 55 transcripts across all NDs depicted in terms of heatmap (Figure 3). The heatmap diagram indicates that 7 genes (i.e., BQ170823, CA746776, CA745229, AJ698954, CA745977, CA747262, and CA744247) are up-regulated in four NDs, which infers that these genes are crucial for different biological processes and metabolic pathways associated with abiotic stresses.

### Singular enrichment analysis of NRGs

**Figure 4:**
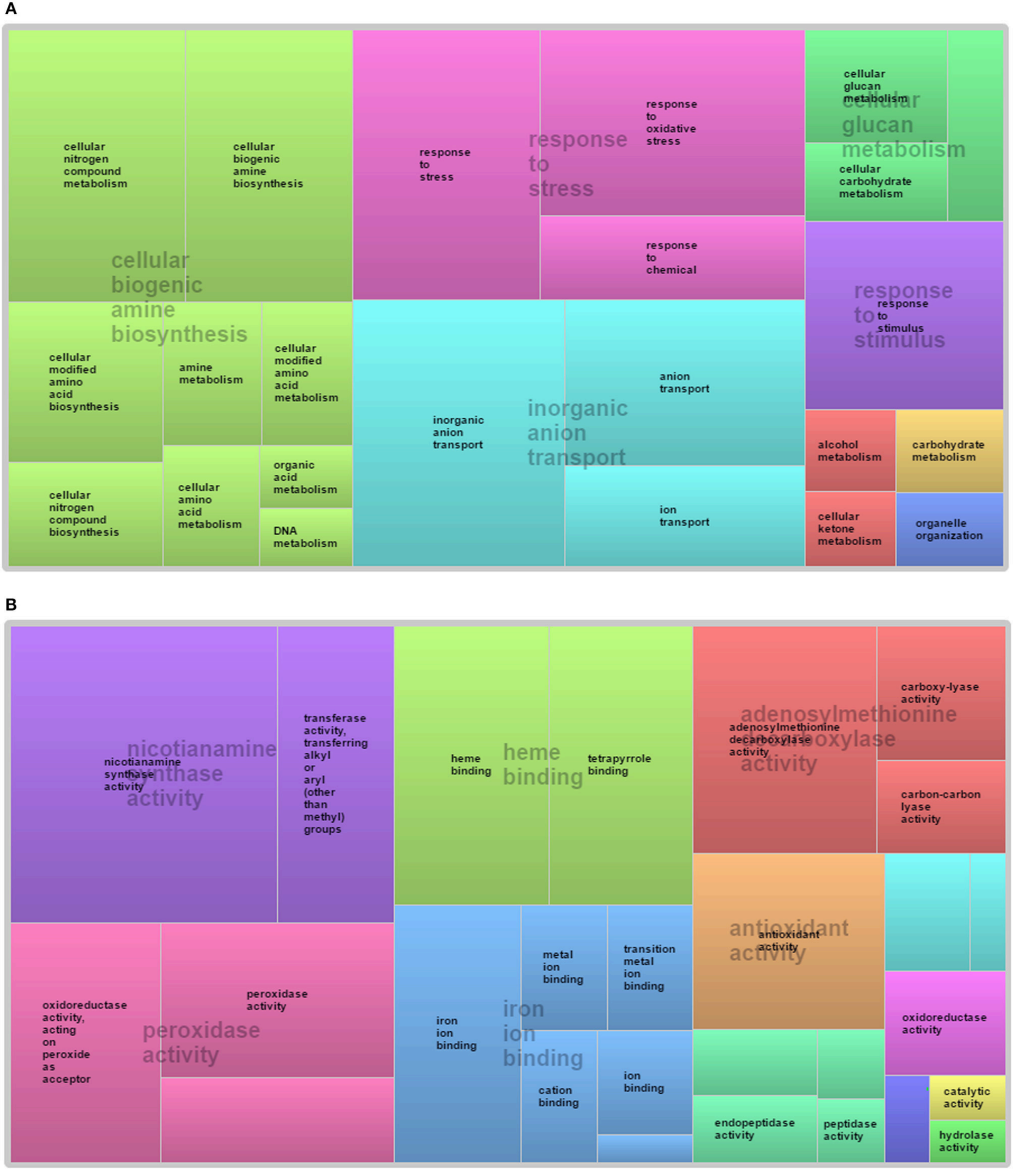
Global Gene ontology based classification of wheat root NRGs in response to K, P, N, Mg, and S deficiency. NRGs participating in different in **(A)** biological process and **(B)** molecular function.

The SEA of 435 probes was executed to annotate their Gene Ontology (GO) terms. Only 291 probes were satisfying the significance criteria (given in material and methods section) and GO term were assigned for each probe (Table S1), while for remaining 144 probes GO terms were not identified (Du et al., 2010). Each probe name was retrieved from Affymetrix®GeneChip®wheat genome Array platform file and revalidated through comparative analysis with Rice and *Arabidopsis* ontology using PLEXdb and ARTRAdb databases. Analysis illustrates that number of NRGs participation in biological processes, molecular function and cellular components decrease according to their sequence. In the biological process, most of the NRGs were classified into cellular process and metabolic process *Viz*. cellular biogenic amine biosynthesis, response to stress, response to stimulus, inorganic anion transport and cellular glucan metabolism (Figure 4A). Within the molecular function category, heme binding, iron ion binding, peroxidase activity, nicotianamine synthase activity, antioxidant activity, oxidoreductase activity, adenosylmethionine decarboxylase activity are the subcategories of GO terms in which genes are playing role (Figure 4B). In cellular component category, a significant number NRGs were classified into vesicle, cytoplasmic membrane-bounded vesicle, membrane and integral to membrane.

### MapMan based pathway analysis

**Figure 5:**
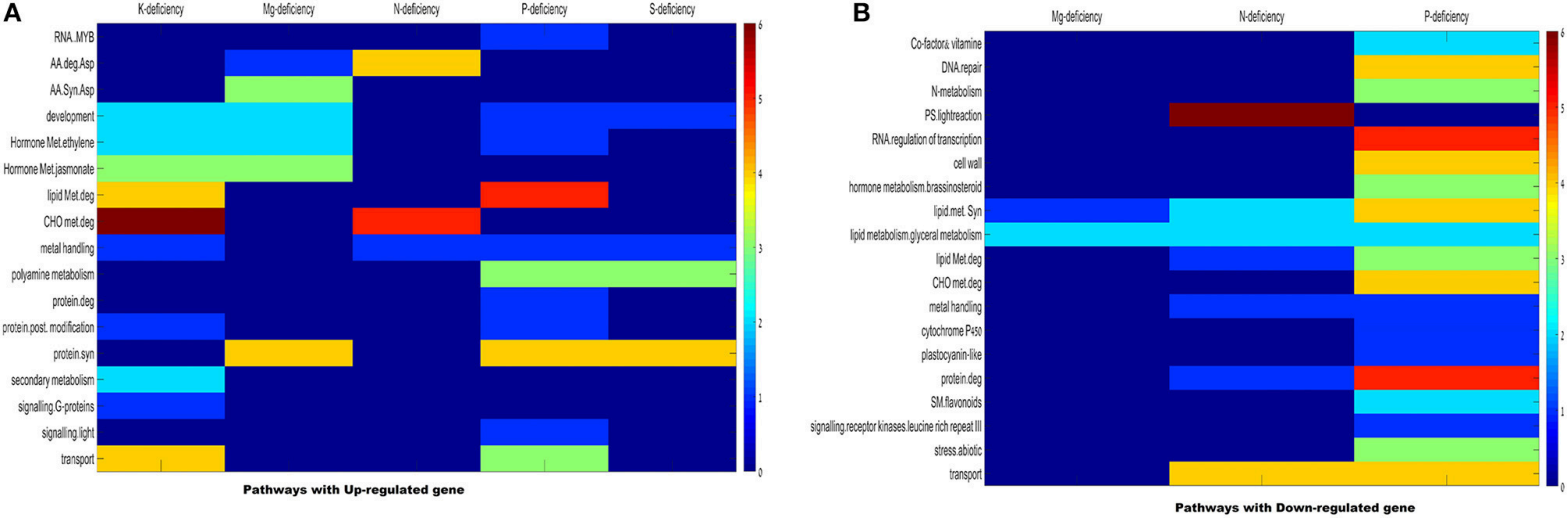
Distribution of significantly enriched pathways from key categories across experiments. The heat map illustrates scaled distribution of the number of pathways significantly enriched. **(A)** Up-regulated genes across key MapMan categories. **(B)** Down regulated genes across key MapMan categories. For each stress condition, the data point with the largest number of pathways was defined as 6 (dark red), to which all other points in the same conditions were scaled against (color bar on right).

To investigate the effect and changes occurs in wheat root due to ND through different metabolic pathways, a MapMan based pathway analysis was performed (Thimm et al., 2004). In this analysis, MapMan wheat genome array file containing BINCODE, name, and description of pathway (http://mapman.gabipd.org/web/guest/mapmanstore) was used to annotate the pathways influenced during different ND conditions using in-house PERL scripts. The mappings of transcriptome data define the functional categories and identify the significant role of different pathways. The plant root expansion is directly related to growth of shoots and formation of booting stages. During these stages, essential macronutrients such as **K, P, S, Mg, N** etc. are gradually accumulated through root. Table 1 shows the name and description of genes participating in different pathways/process with their corresponding hypergeometric *P*-value in Tables S3, S4. Total 110 BICODEs were associated with 398 NRGs and falls within superset of 17 BINCODE and 19 BINCODE or pathway (highly enriched) for up-regulated and down-regulated genes respectively, illustrated in Figure 5. This figure indicates most of the down-regulated genes under the **P** deficiency, participates in metabolic pathways such as carbohydrate (CHO), nitrogen, polyamine, co-factor, vitamin metabolism and synthesis pathways of cell wall, cell wall cellulose, DNA/chromatin, RNA, glycerol, lipid, peroxides, fatty acid and their elongation, although a few of these show active involvement in abiotic stress (i.e., cold, drought and salt), cytochrome P450 pathway and major intrinsic protein transport. Further, the pathway analysis of up-regulated genes under the **P** deficiency inferred their involvement in the transport of phosphate, synthesis of ribosomal protein, polyamine and lipid metabolism, RNA-mediated regulation of transcription and MYB-related transcription factor activity (Figure 5A). The pathways analysis of down-regulated genes under the **N** deficiency indicates their role in Photosystem-I, II, and III light reactions, lipid degradation, glycolipid synthesis and transport of potassium (Figure 5B). Up-regulated genes (CA683846, CK163751) under the **N** deficiency are involved in the reaction mechanism of amino acid metabolism and its degradation, while down-regulated genes (BJ219294, BJ247599) participated in the activation/deactivations of CHO metabolism. The pathways in which up-regulated differentially expressed genes play an important role under the **K** deficiency is CHO metabolism, hormone metabolism, signaling G-proteins, lipid metabolism/degradation and metal handling process (Figure 4A). Gene CA650490 is involved in the signaling pathways of plant defense hormone Jasmonate acid biosynthesis and its degradation (Turner et al., 2002). The ethylene synthesis is involved in the inhibition of cell division, DNA synthesis, growth of the meristems and axillary buds (Burg, 1973). Up-regulated genes (BT00925, BE446498, and CA650490) under **Mg** deficiency participated in amino acid and hormone metabolism while only 3 down-regulated genes (BG908438, BJ307743, and CA725003) are associated with lipid metabolism. Only 6 up-regulated genes under the **S** deficiency are found to be associated with the metal binding process, polyamine metabolism and cellular developments (Table S3). Overall MapMan analysis predicts the metabolic pathways and associated genes plays significant role in survival of plant in response to multiple nutrient deficiencies.

**Table 1:** List of identified significant genes, involved in different metabolic pathways and biological process in response to different nutrients deficiency.

**Gene IDs**	**Genes name/Description**	**Involved in metabolic pathways/Biological process**	**Associated nutrients deficiency**
BT009245	Asparagine synthetase	Amino acid metabolism	Mg
CK163751, CA683846	Asparaginase gene	Amino acid metabolism	N
CA499821,CN012478	RPA1B - Putative single-stranded DNA binding complex subunit 1	RNA regulation of transcription	P
CK211496	Photosystem I protein	Light Reaction of Photosystem	N
CK218048	Photosystem II protein	Light Reaction of Photosystem	N
CA600874	Photosystem-1 F subunit	Light Reaction of Photosystem	N and S
BJ263780	Glutamine synthetase	Nitrogen/ammonia metabolism	P
BE446498	Gibberellin 20 oxidase 2	Hormone metabolism	K and Mg
CA725003	Ubiquitin-specific protease 25	Lipid metabolism	P, N, and Mg
BG908438	Sulfoquinovosyl transferase / Sulfolipid synthase	Lipid metabolism/Glycolipid synthesis/Sulfolipid synthase	N and Mg
CK214660	Fatty acyl coA reductase	Fatty acid synthesis and Fatty acid elongation.	K and P
CA650490	12-oxophytodienoate reeducates	Hormone metabolism, Jasmonate biosynthesis and degradation	K and Mg
CA603808	Receptor-like kinase RHG1	Brassinosteroid signal transduction	P
BQ161477, BQ168403,CA602830	Glycerophosphoryl diester, phosphodiesterase family protein	Lipid metabolism, Fatty acid synthesis and regulation	P and N
CA671780	Cysteine proteinase	Lipid metabolism, Fatty acid synthesis and regulation	N
BQ162635	Nucleoside-triphosphatase	Nucleotide metabolism and degradation	P
CK151676	Adenosylmethionine decarboxylase family protein	Polyamine metabolism	P and S
CK169206	S-adenosyl-l-methionine decarboxylase leader peptide	Polyamine metabolism	P
CA606093	S-adenosylmethionine decarboxylase	Polyamine metabolism	P and S
CA655039, CK158750	CAMK includes calcium/calmodulin depedent protein kinases,	Protein posttranslational modification pathways	N
CD490706	CBL-interacting protein kinase 15	Protein posttranslational modification pathways	N
BJ207672, BJ214953	Serine/threonine-protein kinase	Posttranslational modification	P
AY575717	Monococcum vacuolar invertase1	CHO metabolism	K
BJ219294,BJ247599	Glycosyl hydrolases	CHO metabolism	N
BJ281448,BJ282340, BJ286574,CD878292, CK195601	Nicotianamine synthase	Heme Binding	P, N, K, and S
CD906539	Metallothionein	Metal Binding	N and P
BJ228498,BJ265819	Core histone H2A/H2B/H3/H4 domain containing protein	DNA Synthesis/Chromatin structure modification	P
CA499821, CD453942, CN012478	RPA1B - Putative single-stranded DNA binding complex subunit 1	DNA Synthesis/Chromatin structure modification	P
CD869995	Histone H2A protein	DNA Synthesis/Chromatin structure modification	P
CK200238	Minichromosome maintenance MCM complex subunit 5	DNA Synthesis/Chromatin structure modification	P
CK210306	Methyladenine Glycosylase	DNA Synthesis/Chromatin structure modification	P
CK214714	Core histone H2A/H2B/H3/H4 domain containing protein	DNA Synthesis/Chromatin structure modification	P
CA607301	Nitrate/Chlorate Transporter	Transporter activity	K and N
CD374115	NOD26-like intrinsic protein	Transport,major Intrinsic Proteins	
CA619486	Major facilitator superfamily antiporter	Transport, major Intrinsic Proteins	P
BJ291456, BJ290445	AWPM-19-like membrane family protein	Transport, major Intrinsic Proteins	P
BJ213871	C-5 cytosine-specific DNA methylase	DNA methyltransferases sysnthesis	P
BJ219357	CSLF6-cellulose synthase-like family	Cell wall cellulose synthesis	P
BJ303140	CESA1 - cellulose synthase	Cell wall cellulose synthesis	P
BJ267344	Glutathione S-transferase	Cell wall cellulose synthesis	K and Mg
AY543544	Triticum aestivum beta-expansin TaEXPB5 mRNA, complete cds	Cell wall modification	P
BG907987	Integral membrane HPP family protein	Cell wall modification	N and P
CA606310, CA644687	Glycosyl hydrolases family 16	Cell wall modification	P
CA609165, CA611186, CA638337, CA640998	Ecpension Precorsor	Cell wall modification	P
AJ698954	Tetratricopeptide repeat domain containing protein,	Development, Late embryogenesis abundant	Mg, P, and S
BJ307743	Purple acid phosphatase	Miscellaneous acid and other phosphatases	P, Mg, and N
CA648984, CD889338, BJ292311	Full length mRNA Sequence/ Cytochrome P450	Miscellaneous, Cytochrome P450 regulation	P, K and N
CA722714, CA718376, CD909074	Dehydrin family protein	Drought, cold, and salinity stress	N, P, and K
BJ298679, BJ299408	Late embryogenesis abundant protein	Drought, cold, and salinity stress	N and P
BJ286151	Peroxidase	Active role in detoxifying ROS during stress	N and P
BJ305885	SPX domain containing protein	Phosphate homeostasis	N, P, and Mg
CA745229	78 KDA Glucose-Regulated Protein	Defense mechanism	N, P, Mg, and S
CD909599	Transcription activator-related protein	Transcription	N and P
CA638726, BQ170823, BQ788879, CA606888, CA745977, CA747262, BQ165954, CA747319, CA638102, CA596310, CD490483, CA744694, CA745341, CA688900, CA744247, CA606590, CA744333, CD901150, CA746776, CA611390	Transcribed gene sequences	Not identified	N, P, K, S, and Mg
CK214709	Hypothetical protein	Not identified	K, Mg

*The hypergeometric P values of a gene/genes group is shown in Table S4.

### Meta-analysis of NRGs

**Figure 6:**
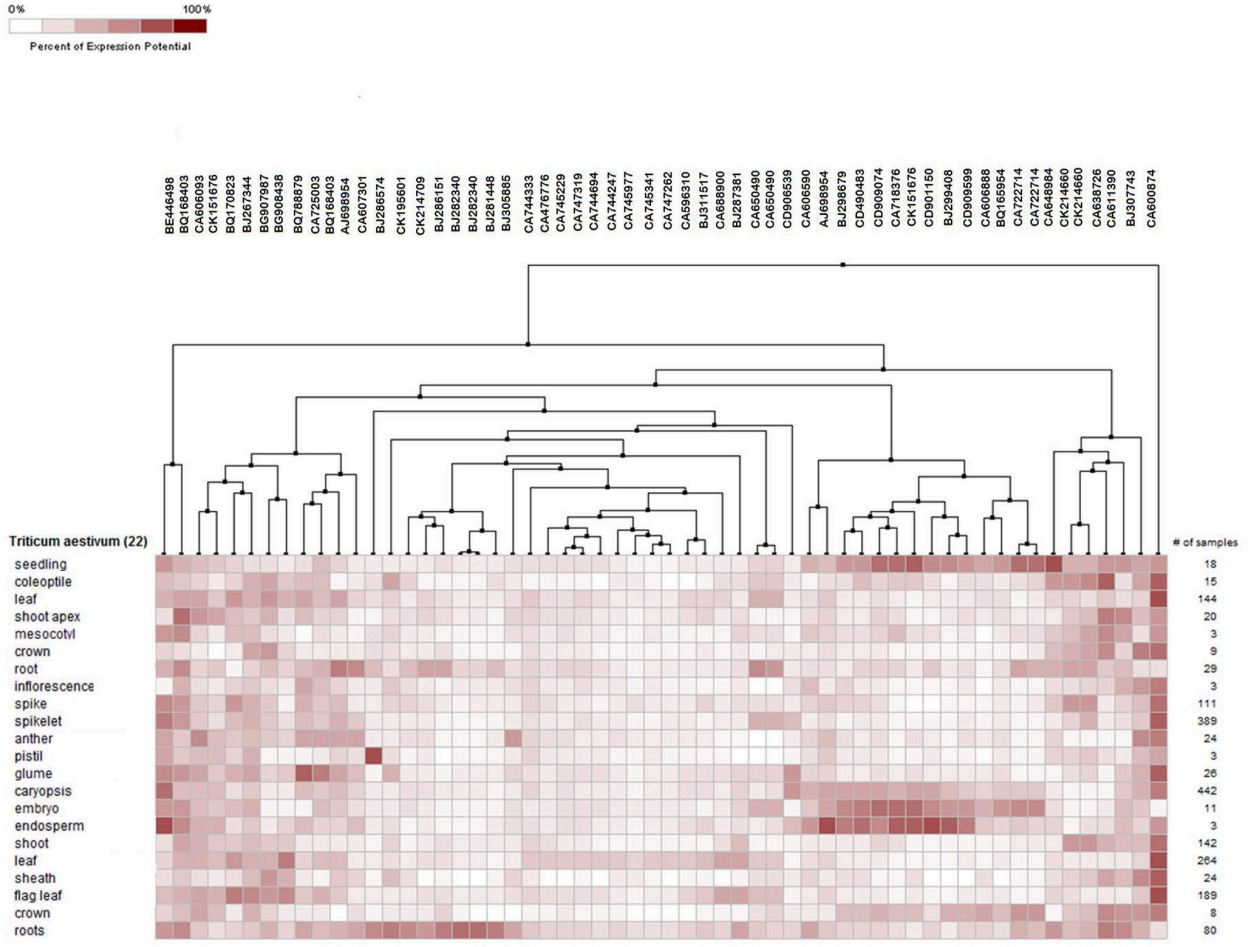
Tissue specific potential gene expression of 55 NRGs against selected root samples. The expression potential of the genes in a tissue of wheat is measured in term of percentages value, which is shown in color key.

**Figure 7:**
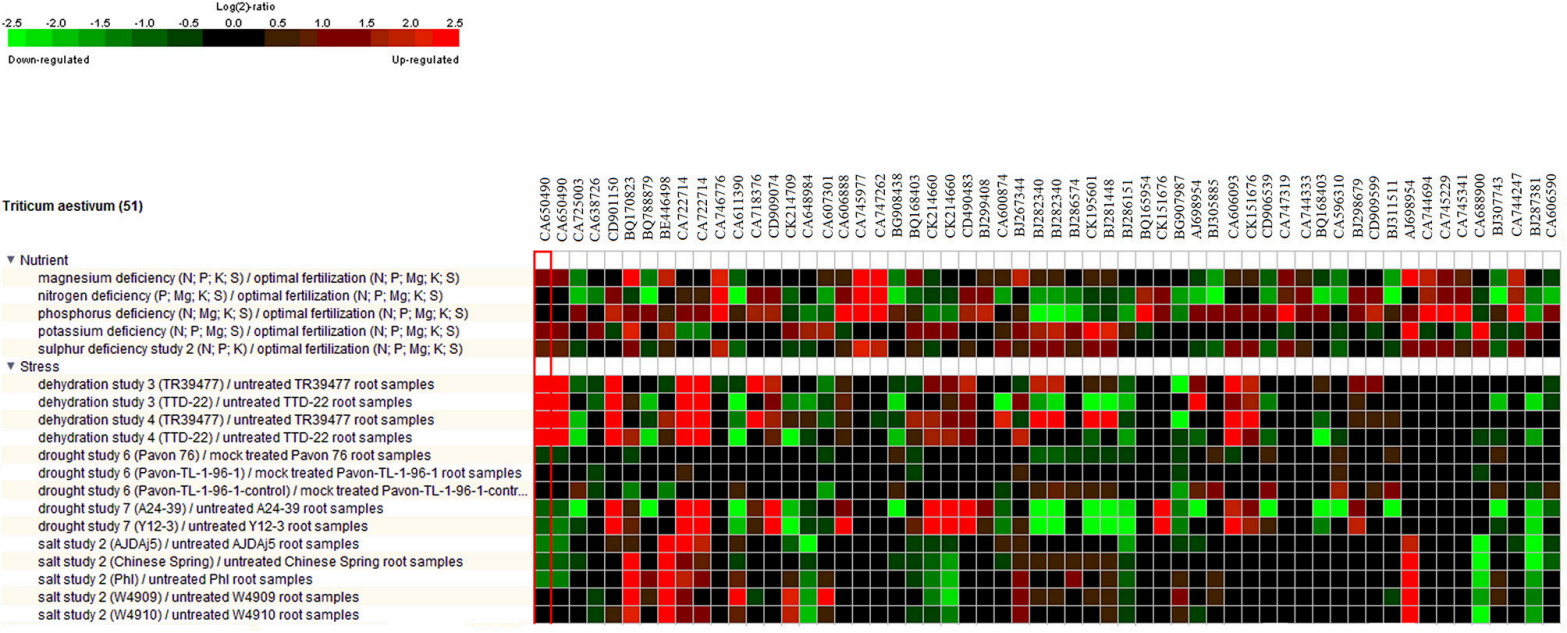
Heat map diagram for 55 selected NRGs while comparing with root specific samples. The green and red colors specify down-regulation (log2 [2.5]) and up-regulation (log2 [−2.5]), respectively as shown in the color bar. Similarity search was done using subsets of individual stress and nutrient deficiency conditions such as heat, cold, drought/dehydration, salt, submergence, and shift from aerobic to anaerobic germination cold and drought. The expression data were obtained using Genevestigator (Zimmermann et al., 2008).

Meta-analysis provides a significant analysis of the genes of interest based on a large-scale systematic combination of normalized and quality-controlled expression data with experimental context variables using different ontologies i.e., anatomy development, perturbation, or genetic background (Hughey and Butte, 2015). Meta-analysis of NRGs was performed using the Genenvestigator tool, which has wild type and non-wild type 2101 samples of Affymetrix®GeneChip®Wheat Genome Array in its knowledgebase. These samples were generated under 164 different conditions in seedling, inflorescence, shoot and rhizome across the world. The root specific 109 samples for single experiment analysis and compendium wide analysis was selected (Table S5) for the said meta-analysis. Single experiment analysis annotates the percentage expression of each probe across the root specific wheat samples. The percentage of gene expression potential of each NRGs across the selected 109 root specific samples were generated through Single experiment analysis (Figure S3). Figure S3 indicates that large numbers of NRGs were differentially expressed while wheat root expose to NDs, biotic and abiotic stress and dehydration. Further, the compendium analysis facilitates gene expression intensity on a particular tissue with their favorable conditions of gene regulation. A hierarchical clustering method using Euclidean distance function was used to annotate the tissue-specific expression with respect to all samples (Hruz et al., 2008). Clustering of all probes using Genevestigator reveals the level of expression patterns of NRGs in 22 different tissues of the wheat (Figure S4), which is very crucial to identify the function of particular genes. The higher level of expression of a candidate gene in particular tissue will be helpful in inferring its role. Similarly, tissue based expression potential of 58 probes playing a crucial role in at least two ND conditions were also annotated and depicted in Figure 6. Moreover, in compendium analysis, the signature tool was used to perform comparative analysis in order to identify other stress conditions, which causes the similar gene expression signature using selected samples. The signature tool uses selected standard datasets, to find out whether the NRGs expression trend in selected samples (at a given condition) is similar or contradictory to the expression trend of standard datasets (Figure S5). Similar analysis was also performed for 58 NRGs and same condition is observed as in figure Figure S5. The comparative expression potential of 55 genes in stress responsive conditions and is shown in Figure 7. Further, it has been observed out of 398 NGRs, only 137 genes are found to encode the protein sequences, which were annotated using standard sequence analysis protocols. Out of 137 proteins, 42 characterized and 95 uncharacterized proteins were identified (Table 2). In addition, the expression signature of these proteins in specific macronutrient deficiency conditions were termed as “Low,” “High,” and “Medium” (Table 2). These terms were calculated according to respective color values obtained through Genevestigator tool, as shown in Figure 7. Taking into the account of NDs conditions, the scale “low” is denoted = 3 out of 5 experiments were represented with black color. Similarly for “Medium” = 2 and for “High” = 1 out of 5 black color representations were considered in ND. Likewise in stress conditions = 8 out of 14 were termed as “Low,” whereas = 5 was considered as Medium and = 2 as High respectively (the black color box indicates that gene is not expressed in respective condition). Similar analysis was also performed for other stress conditions and the results are summarized in Table 2.

**Table 2:** List of differentially expressed probes and encoded protein description identified through Meta-analysis.

**Probes**	**Uniprot ID**	**Description of proteins/enzymes**	**Expression in ND conditions**	**Expression in stress conditions**
**CHARACTERIZED PROTEINS/ENZYMES**				
Ta.254.1.S1_s_at	Q9FVM8	Cytochrome P450	Low	Low
Ta.4936.1.S1_at	W5EUQ8	Non-specific serine/threonine protein kinase	Medium	Medium
Ta.28002.1.A1_at	W5DXD9	Non-specific serine/threonine protein kinase	Medium	Low
Ta.25609.1.S1_at	W5ETB2	Non-specific serine/threonine protein kinase	Medium	Low
Ta.2870.1.S1_at	Q6RUJ1	Glutamine synthetase	Medium	Low
Ta.27657.12.S1_at	W5H2P2	Histone Protein	Medium	High
TaAffx.82031.1.S1_s_at	W5AB05	Peroxidase	Medium	High
Ta.9451.1.S1_x_at	W5CNW4	Peroxidase	Low	Medium
Ta.24687.1.S1_at	W4ZNA8	Peroxidase	Low	Medium
Ta.2746.1.S1_x_at	W5I3P6	Peroxidase	Low	High
Ta.14580.1.S1_at	W4ZR05	Peroxidase	Low	Medium
Ta.4928.1.S1_at	W5A1N2	Peroxidase	Low	Low
Ta.5406.1.S1_at	W5A1N2	Peroxidase	Low	Low
Ta.5576.2.S1_a_at	W5HQZ7	Peroxidase	Low	Medium
Ta.952.2.S1_a_at	W5HZ76	Peroxidase	Low	Low
Ta.14580.3.S1_x_at	W4ZSN6	Peroxidase	Low	Medium
Ta.5640.2.A1_x_at	A0A096UN06	Peroxidase	High	High
Ta.2746.3.S1_x_at	W5HKU0	Peroxidase	Medium	Medium
Ta.29637.1.S1_at	W5A9M2	Peroxidase	Medium	Medium
Ta.1807.1.S1_at	W5BMF0	peroxidase	Medium	Medium
TaAffx.111224.1.S1_at	W5BMF0	Peroxidase	Medium	High
Ta.8805.1.A1_at	W5BJ15	Peroxidase	Low	Medium
Ta.13811.1.S1_at	G9DRA4	MYB-related protein	Medium	Medium
TaAffx.4871.1.S1_at	W5FM16	Kinesin-like protein	Medium	Low
Ta.5652.1.S1_at	A7J2I4	Tonoplast intrinsic protein	Low	High
TaAffx.84102.1.S1_at	W5I0I5	Xyloglucan endotransglucosylase/hydrolase	Medium	High
Ta.9396.1.S1_a_at	W5CS03	Xyloglucan endotransglucosylase/hydrolase	Low	Medium
Ta.18791.1.S1_at	W5I0I5	Xyloglucan endotransglucosylase/hydrolase	Low	High
Ta.2690.1.S1_at	Q5QFC3	Glutamine-dependent asparagine synthetase (ASN1)	Medium	Medium
Ta.216.1.S1_at	Q9ZR84	Pollen allergen homolog	Medium	High
Ta.9063.1.S1_x_at	W5GZ28	S-adenosylmethionine decarboxylase proenzyme	Medium	Medium
Ta.9063.2.A1_at	W5GKA3	S-adenosylmethionine decarboxylase proenzyme	Medium	Low
Ta.113.1.S1_at	Q41515	Potassium Uptake transporter (hkt1)	High	Low
Ta.23659.1.S1_at	AAN74638	LEA Proteins	Medium	Low
Ta.30668.1.S1_at	Q6QF95	Expansin EXPB10	Low	Medium
TaAffx.60291.1.S1_at	W5GG94	Cellulose synthetase	Low	Medium
Ta.19183.2.S1_at	W5FDE6	CASP-like protein	Medium	High
Ta.27027.1.S1_at	A5JPR1	Leucine-rich repeat protein (LRR2)	Medium	High
TaAffx.111940.1.S1_s_at	AAC23502.1	Vacuolar invertase	Low	Low
Ta.28802.3.S1_at	W5FFS4	Histone H2A Protein	Low	High
Ta.2148.1.S1_x_at	Q9LKM4	Cold-responsive protein (Wlt10)	Low	Low
Ta.18487.1.S1_x_at	Q9LKM4	Cold-responsive protein (Wlt10)	Low	High
**UNCHARACTERIZED PROTEINS/ENZYMES**				
Ta.975.2.S1_at	W5D848	Glutaredoxin family protein	Low	Low
Ta.26280.1.S1_at	W5DJG7	AAA-type ATPase family protein	Low	Low
Ta.8188.1.S1_at	W5C672	Transcription factor bHLH54	Low	Medium
Ta.11367.2.S1_at	A0A077RTB3	Protein serine\threonin kinase activity	Low	Medium
Ta.21039.1.S1_at	W5G3R0	Retrotransposon protein	Low	Medium
Ta.5148.1.S1_a_at	W5E2J6	Xylem cysteine proteinase 2 precursor	Medium	Medium
Ta.5148.2.S1_x_at	W5F844	Fruit bromelain	Medium	High
Ta.5148.3.S1_x_at	W5E6K3	Fruit bromelain	Low	Medium
Ta.14580.2.S1_at	W5AGX1	Peroxidase precursor	Low	Medium
Ta.8808.1.A1_at	W5GVT9	C2 domain-containing protein-like	Medium	Medium
Ta.5538.1.S1_at	W5GY14	Cupin domain containing protein	Low	Medium
Ta.28528.1.S1_at	A0A077RVV7	Cupin domain containing protein	Medium	Medium
Ta.18318.1.S1_at	W5E6B2	Annexin	Low	High
TaAffx.69460.1.S1_at	W5DWK4	Protein dna J	Low	Low
Ta.9143.1.S1_at	W5A632	NOD26-like intrinsic protein	Low	Low
Ta.7978.1.A1_at	W5G5Z0	Transcription factor MYB39	Low	Medium
TaAffx.53797.1.S1_s_at	W5GBK8	MYB-related protein	Low	Low
Ta.18779.1.S1_at	W5BBI0	Beta-fructofuranosidase, insoluble isoenzyme 7	Low	High
Ta.20483.1.S1_at	W5ERW2	Dehydrin	Low	Low
Ta.5844.1.S1_at	W5GD49	Dehydrin DHN3	Medium	Low
TaAffx.46097.2.S1_at	W5F2F6	Dehydrin 2	Low	Medium
TaAffx.91995.1.A1_at	W5BGS8	Prolyl endopeptidase	Medium	High
Ta.23191.1.S1_at	W5BQ68	S-layer protein	Low	Medium
TaAffx.119251.1.S1_s_at	A0A077S321	Metal ion binding protein	Low	High
Ta.27970.1.A1_at	W5H5C0	Farnesylated protein 1	Low	Medium
Ta.23068.1.S1_at	W5BTL8	S-norcoclaurine synthase	Medium	Medium
Ta.5734.1.S1_at	W5B5J8	Protein phosphatase 2C	Low	Low
Ta.3618.1.S1_at	W5G7H1	DNA helicase	Low	Low
Ta.9600.1.S1_x_at	W5FTZ3	Early light-inducible protein HV90	Low	Low
Ta.26997.1.S1_at	W5EH42	Early light-induced protein	Medium	Medium
Ta.14587.1.S1_at	W5I132	HMG transcription factor	Low	Medium
Ta.9564.1.S1_at	W5FU79	Universal stress protein	Low	Low
Ta.1207.1.S1_at	W5HU75	12-oxophytodienoate reductase 2	Medium	Medium
Ta.5388.1.S1_a_at	W5CU17	Caffeic acid 3-O-methyltransferase	Medium	High
TaAffx.59372.1.S1_at	W5FZG1	Lysosomal beta glucosidase	Low	Low
Ta.9029.1.A1_at	A0A096UTP6	PE_PGRS54	Low	Low
TaAffx.29542.1.S1_at	W5E034	ABC transporter G family member 16	Low	Medium
Ta.15889.3.A1_a_at	W5GX99	Cytosine-specific methyltransferase	Medium	Medium
Ta.19715.1.S1_at	W5HWS7	Phosphate transporter 1	High	Medium
Ta.9063.3.S1_at	W5GBL0	S-adenosylmethionine decarboxylase proenzyme	Low	Low
Ta.9063.3.S1_x_at	W5GBL0	S-adenosylmethionine decarboxylase proenzyme	Low	Low
Ta.6416.1.S1_at	W5CE21	Glycerol-3-phosphate acyltransferase	Low	Medium
Ta.556.1.S1_at	W4ZWM7	Lipase proteins	Medium	Medium
TaAffx.51582.1.S1_s_at	W5BH10	Early nodulin-like protein 2	Low	High
TaAffx.37775.1.A1_s_at	W5G9S0	NAC domain-containing protein	Low	Medium
TaAffx.106421.1.S1_at	W5B7T3	Zinc transporter	Low	Medium
TaAffx.52861.1.S1_at	A0A077RZR2	Zinc finger DNA-binding domain	Low	Low
Ta.4210.3.S1_at	W5FTM2	Pyruvate dehydrogenase E1 component subunit beta	Low	Medium
Ta.6253.1.S1_a_at	W5HYL7	Cylin protein	Low	Low
Ta.6770.1.S1_s_at	W5BU24	Glycerophosphoryl diester phosphodiesterase family protein	Low	Low
Ta.23095.1.S1_at	W5CHA3	Glycerophosphodiester phosphodiesterase GDE1	Medium	Low
TaAffx.12868.1.A1_at	W5B703	Putative glycerophosphoryl diester phosphodiesterase 1	Low	High
Ta.24434.1.S1_at n	W5BU24	Glycerophosphodiester phosphodiesterase GDE1	Low	Low
Ta.22083.1.S1_at	W5EQG4	Glycerophosphodiester phosphodiesterase GDE1	Low	Low
Ta.26144.1.A1_at	W5HE25	Male sterility protein	High	Medium
TaAffx.29217.1.S1_at	W5B5T8	Disease resistance response protein	Low	Medium
TaAffx.12181.1.S1_at	W5G1I5	Bidirectional sugar transporter SWEET17	Low	Medium
Ta.10768.1.A1_at	W4ZLL9	Protein RUPTURED POLLEN GRAIN 1	Low	Low
Ta.13993.1.S1_x_at	W5BPQ3	SPX domain-containing protein 6	Medium	High
TaAffx.104994.1.S1_at	W5HRB6	Putative iron-deficiency specific 4 protein	Medium	High
TaAffx.115546.1.S1_at	W5BPQ3	SPX domain-containing protein 6	High	High
Ta.16683.1.A1_at	W5EQW2	BURP domain containing protein	Low	Medium
TaAffx.3993.1.S1_at	W5EA81	DUF260 domain containing protein	Low	Low
Ta.24423.1.S1_s_at	W5E611	Triticum beta-expansion	Low	Medium
Ta.7592.1.S1_at	W5HYY5	CSLF6 - cellulose synthase-like family F	Medium	High
TaAffx.72706.1.A1_at	W5E830	Nonspecific serine/threonine protein kinase	High	Medium
Ta.3093.1.S1_at	W5EH36	Replication protein A 70 kDa DNA-binding subunit	Low	Medium
Ta.3093.2.A1_at	W5E0S9	RPA1B - Putative single-stranded DNA binding complex subunit 1	Low	Low
Ta.3093.3.A1_at	W5EH36	Replication protein A 70 kDa DNA-binding subunit	Low	Medium
Ta.5843.1.S1_x_at	W5E046	Late embryogenesis abundant protein (LEA)	Low	Medium
Ta.23812.1.S1_a_at	W5AA10	AWPM-19-like membrane family protein	Low	Low
Ta.28555.2.S1_at	W5FSN3	AWPM-19-like membrane family protein	Low	Low
Ta.30509.1.A1_at	W5ESS7	Permease	Low	High
Ta.7871.2.S1_a_at	W5D9W0	Glycerol-3-phosphate dehydrogenase	Low	Medium
Ta.1464.2.S1_a_at	W5DID9	Major facilitator Superfamily antiporter	Low	Medium
Ta.8850.1.A1_x_at	W5HWS2	HIPL1 protein	Low	High
Ta.30950.1.A1_x_at	W5C241	Glycosyl hydrolases	Low	Low
Ta.25277.1.A1_at	W5BD82	Beta-fructofuranosidase, insoluble isoenzyme 7	Low	Medium
Ta.1783.1.S1_at	W5ENU1	Cysteine protease	Low	Medium
Ta.667.1.A1_at	W5EQZ6	Integral membrane HPP family protein	Medium	Low
TaAffx.29848.1.S1_at	W5GEK7	Kinesin-like protein	Medium	Medium
TaAffx.24477.1.S1_at	W5EZH8	Expressed protein	Medium	Medium
TaAffx.124214.1.S1_at	W5F7L0	Chromatin assembly factor 1 subunit FSM	Medium	Medium
Ta.937.1.A1_at	W5AMZ2	Hydrolase, alpha/beta fold family protein	Low	Medium
TaAffx.19033.1.S1_at	W5A2N8	TPR doamin protein	Low	Low
Ta.14998.1.S1_at	W5A222	TPR repeat-containing thioredoxin TTL1	Low	High
Ta.3784.2.S1_at	W5E8I6	Protein IN2-1-like protein B	Low	Low
Ta.25642.1.A1_at	W5DLK3	Terpene cyclase/mutase family member	Low	Medium
TaAffx.59304.1.A1_at	W5DWS3	Leucine Rich Repeat family protein	Low	High
Ta.1840.1.S1_at	W5B904	Protease inhibitor-like protein	Low	High
Ta.1840.2.S1_x_at	W5AV07	14 kDa proline-rich protein	Low	Medium
Ta.5435.1.S1_x_at	W5H9Y6	LTP family protein precursor	Low	Medium
Ta.13950.1.S1_x_at	W5HAD3	Cortical cell-delineating protein	Medium	Medium
Ta.14492.2.S1_at	W5E7A4	Cortical cell-delineating protein	Medium	High

*Result of Compendium Wide analysis provides the expression level of a probe across selected 109 samples of root in response different stress. Level of gene expression measured in specified experimental sets termed as “Low,” “Medium,” and “High” as shown in Figure 7. Taking into the account of ND conditions, the scale “Low” is denoted ≥3 out of 5 experiments were represented with black color. Similarly for “Medium” ≥2 and for “High” ≤ 1 out of 5 black color representations were considered in ND. Likewise in stress conditions ≥8 out of 14 were termed as “Low,” whereas ≥5 was considered as Medium and ≤ 2 as High respectively (the black color box indicates that gene is not expressed in respective condition)*.

### Expression and functional analysis of transcript encoding key characterized enzymes involved in ND and stress

ND conditions generate adverse environmental growth conditions for plant, which results in inducing the degradative processes. During such conditions/treatments, plant cells acquire ability to respond to these diverse stresses by the means of various flexible and balanced stress response networks. These networks involves various metabolites such as reduction-oxidation (redox), stress hormones, signaling pathways, reactive oxygen species (ROS), starch biosynthesis and growth regulators, as well as calcium and protein kinase cascades etc. (Gill and Tuteja, 2010; Foyer et al., 2016; Gill et al., 2016; Batra et al., 2017). In our study, 16 transcripts were found to encode peroxidase family enzymes (EC 1.11.1.7), which is an oxidoreductase that uses several inorganic and organic substances as hydrogen donors in the presence of H_2_O_2_ (Dawson, 1988; Siegel, 1993). Generally, peroxidase enzymes are located in the cell wall of plant cells and act as a crucial enzymatic system involved ROS generation (Bela et al., 2015; Camejo et al., 2016). High expression of peroxidase family enzymes indicates that it used soil inorganic and organic substances to provide the required energy to plant during low nutrient treatment of wheat root (Francoz et al., 2015). Further analysis suggested that 3 transcripts encode plant cell wall enzyme xyloglucan endotransglucosylase/hydrolases (XTH), which is capable of joining xyloglucan chains to oligosaccharides. XTH (EC 2.4.1.207) is involved in the alteration of the load-bearing cell-wall components and these properties make it very important in the regulation of growth and development of shoot and root (Table 2; Maris et al., 2011; Hara et al., 2013). Expression of XTH transcripts during ND condition affect the growth of wheat root as the earlier study by Maris et al. in *Arabidopsis* root also corroborate with our finding (Maris et al., 2009). The up-regulated expression of the transcript, code Plant Potassium Transporter (HKT1) indicates the transportation of the **K** in plant cells is required for a wide variety of functions like maintenance of electrical potential gradients across cell membranes, generation of turgor and activation of numerous enzymes. Most of these functions directly depend on the transportation of potassium concentration across membrane-bound K transport proteins (Britto and Kronzucker, 2008). Interestingly, 2 transcripts expression encode cold responsive protein that indicates that Wlt10 gene of the wheat root is activated during ND condition, which enables root to survive at freezing temperatures (Thomashow, 1998; Gupta et al., 2015a). The expression of S-adenosylmethionine decarboxylase proenzyme (SAMDC, EC 4.1.1.50), as a key rate-limiting enzyme in the polyamine (PA) biosynthesis is required for plant growth and development (Hu et al., 2005). Moreover, PA works as a metabolite and play protective as other defensive roles in response to stress (Liu et al., 2015). Three transcripts encode nonspecific serine/threonine protein kinase enzyme (EC 2.7.11.1) which belongs to the family of transferases (Prasad et al., 2013; Gupta et al., 2015b). Generally, these enzymes are involved in plant response to abiotic stresses and abscisic acid (ABA)-dependent plant development through phosphorylation process (Kulik et al., 2011). Expressions of Late embryogenesis abundant protein (LEA) have been linked to the survival of the wheat root under the intervals of ND and stress through defensive enzymatic function (Gupta et al., 2016a). In spite of decades of effort, the molecular-level mechanisms defining this protective function remain unknown for this protein so far (Amara et al., 2014). Moreover, some other transcripts encode characterized proteins/enzymes such as pollen allergen, cytochrome P450, vacuolar invertase, glutamine-dependent asparagine synthetase, MYB-related protein, leucine-rich repeat protein, tonoplast intrinsic protein, glutamine synthetase, and histone H2A protein play various important roles in ND and stress conditions.

### Expression and functional annotation of transcript encoding key uncharacterized enzymes involved in ND and stress

Functional analysis of uncharacterized proteins was performed on the basis of similarity search against protein database, i.e., PDB, NCBI, and UniProt as well as conserved domain-based protein family annotations (Gupta et al., 2016b). Thus, we have annotated and identified the functions for 106 uncharacterized proteins expressed during different ND. The uncharacterized enzymes with similar sequence to characterize proteins tend to have a similar function, which is already explained characterized enzyme section. The functional analysis of remaining uncharacterized proteins/enzymes, expressed moderately or high during ND and stress conditions are described (Table 2). It was reported that, glycerophosphodiester phosphodiesterase enzyme (GPX-PDE, EC 3.1.4.46) catalyzes the hydrolysis of deacylated phospholipid of glycerophosphodiester to glycerol-3-phosphate and corresponding alcohol. In plant*s*, this enzyme has been characterized through microarray analysis and various studies showed enhanced expression of its transcripts (Uhde-Stone et al., 2003; Misson et al., 2005; Morcuende et al., 2007). Similarly, in our study, up and down-regulated expression of 3 transcripts was observed, which encodes GPX-PDE under the **P** deficiency while in the **N** deficiency down-regulated 2 transcripts encodes GPX-PDE. It plays important role in hair growth and development and in the **P** deficiency induced phospholipid degradation pathway in wheat roots and also found in *Lupinus albus* (Cheng et al., 2011). AWPM-19-like membrane family protein encoded by 2 transcripts in our study plays important roles in plant development and various stress responses (Chen et al., 2015). It has a similar sequence of LEA protein, which is induced by ABA during freezing tolerance (Koike et al., 1997).

## Discussion

### Transcriptomic and meta-analysis of array data of *T. aestivum* cv. root

Since past one and half decade several transcriptome analysis were performed in different plant species in response to diverse micronutrient deficiency to mine the important genes along with their role in different metabolic pathways (Wasaki et al., 2003, 2006; Li et al., 2010a,b; Cai et al., 2012; Ma et al., 2012; Park et al., 2012; Takehisa et al., 2013, 2015; Yu et al., 2016; Forieri et al., 2017; Gupta et al., 2017). In continuation, the current study reports 398 differentially expressed genes through a comparative transcriptomic study of micronutrient deficient samples grown in soil having environment growth conditions. The study identified 58 probes (55 genes) are commonly expressed under at least two ND conditions (Figure 3). The differentially expressed genes were identified through normalization, QC, QA of array data (Raychaudhuri et al., 2000; Bolstad et al., 2003). Further, the GO-based analysis of differentially expressed NRGs mimics their association with distinct GO terms. Through the GO term it can be interpreted that; one NRG can participate in different biological functions and vice-versa (Gupta et al., 2016a). Accordingly, the study identified 291 NRGs associated with different GO terms indicating their roles in regulation of different reaction mechanisms in response to macronutrients stress (Table S1). Taking an example: the peroxidase precursor gene, which generally participate in peroxidase activity (GO: 0004601) also plays an important role in peroxidase reaction (GO: 0006804) in cytoplasmic membrane-bounded vesicle (GO: 0016023) of plant cells. Figure 4 represents different significantly enriched GO terms (*P*-value = 0.05) obtained through functional characterization of NRGs involved in biological processes and molecular functions. The major affected biological processes are cellular biogenic amine biosynthesis, inorganic anion transport, response to stress, response to stimulus and cellular glucan metabolism. 17 NRGs involved in the biosynthesis of cellular amines and poly amines, are found to play a crucial role in different signaling networks and metabolic process of plants (Hussain et al., 2011; Berkowitz et al., 2016). Five NRGs which are involved in the movement of inorganic anions i.e., in or out of the cell or within a cell, or between plant cells are being observed as transporters which indicates their important functions during P, N, and K deficiency (Sharma, 2016; Dong et al., 2017; Forieri et al., 2017). Forty NRGs are participating in response to stress and stimulus indicates their involvement in different metabolic and signaling pathways. Eleven NRGs are found to be involved in peroxidase reaction and oxidative stress. The biosynthesis of glucan/carbohydrate and its metabolism are the key processes mediating plant responses to abiotic stresses. Under such stress, plants generally remobilize glucan/carbohydrate to supply energy and carbon at times when photosynthesis process may become limited (Thalmann and Santelia, 2017). Nicotianamine synthase activity (13 NRGs) and metal ion binding activity (16 NRGs) are the main sub category under molecular functions. Nicotianamine is an intermediate for the biosynthesis of mugineic acid-family phytosiderophores in the crop plant and work as key substance for iron metabolism. Nicotianamine synthase catalyzes the formation of nicotianamine from S-adenosylmethionine (Higuchi et al., 1996). Heme and tetrapyrrole binding may occur during the transportation of the macronutrients for plant organs. Similar GO based analysis was also reported in earlier studies (Ma et al., 2012; Takehisa et al., 2013, 2015; Yu et al., 2016; Shi et al., 2017; Zhu et al., 2017), using the ontological terms to describe the function of differentially expressed genes.

The NRGs were mapped through MapMan scheme (Figure 5) and results indicate the number of significant genes, which are associated with different metabolic pathways in macronutrients deficiency. The study was performed with an assumption that the cellular processes and metabolic pathways involved in supply of water, nutrients factors are in general common for availability and deficiency of **K, N, P, Mg**, and **S**. Interestingly, the metabolic pathway and cellular process are also involved in amino acid metabolism, protein synthesis, metal binding, hormone synthesis, lipid synthesis and its degradation regulated by similar genes under most of the ND cases. Different cellular metabolic processes, i.e., DNA synthesis/repair, RNA regulation and transcription of transcription factors and related proteins, nitrogen/ammonia metabolism, abiotic stress and cell wall synthesis have been found to be affected under the **P** deficiency. Similarly Photosystem process and related light reaction have been found to be affected in the **N** deficiency (Table S4). Further, a meta-analysis of each transcript was performed to identify the differential expression in response to particular ND and stress condition. This analysis summarized the results of single experiment analysis and compendium wide analysis by taking consideration of all 109 wheat root specific microarray data samples using Genevestigator tool (Thimm et al., 2004). Meta-analysis checks the expression potential of each NRG under **Mg, N, P, K**, and **S** deficiency and other stress such as dehydration, drought and salt etc. (Figure 7). Moreover, the hierarchical clustering based tissue-specific percentage expression potential of each transcript explains the tissue-specific role of candidate genes (Figure S4). Further functional role of different NRGs encoding uncharacterized proteins/enzymes (Table 2) were identified and explained. Thereafter, the functional roles of 35 transcripts out of 55 transcripts were annotated through standard protocols (Gupta et al., 2016b). Remaining 20 transcripts are yet to be functionally characterized in wheat varieties, through *in-vitro* validation studies.

### Comparative analysis with transcriptome studies performed *in-vitro* in model plants

Recently, Ruan et al. (2015) performed *in-vitro* transcriptome studies in seedlings of two wheat genotypes i.e., low-**K** tolerant “Tongzhou916” and low-**K** susceptible “Shiluan02-1” using Affymetrix®GeneChip®analyzer in response to low **K** deficiency. They found 2713 and 2485 differentially expressed genes in Tongzhou916 and Shiluan02-1, respectively. Among 2713 genes of Tongzhou916, 1321 genes are up-regulated and 1392 genes are down-regulated; whereas out of 2485 genes of Shiluan02-1, 1177 genes are up-regulated and 1308 genes are down-regulated (Ruan et al., 2015). While comparing with the present study involving differentially expressed genes under **K** deficiency, only 42 and 3 genes are found to be up-regulated and down-regulated respectively. Comparative analysis showed that probes i.e., Ta.21127.1.S1_at, Ta.1207.1.S1_x_at, or Ta.1207.1.S1_s_at encoded for Nitrate/Chlorate transporter (Gene ID: CA607301), and OPR1 12-oxophytodienoate reductase (Gene ID: CA650490), which were commonly expressed in both the study. It indicates their role as the **K** deficiency affected genes, in different stages of wheat growth. Despite this fact, we observed commonly affected metabolic pathways i.e., Jasmonic acid and related, ethylene-related, transporter of the peptide, oligo-peptides, and potassium are affected in both soil-grown and *in vitro* grown wheat roots and seedlings under the **K** deficiency. Moreover, two earlier studies by Armengaud et al. and Ma et al. in model plant Arabidopsis and Rice under the **K** deficiency respectively, also corroborates the role of jasmonic acid signaling (Armengaud et al., 2004; Ma et al., 2012). *In-vitro* transcriptomic studies performed (Li et al., 2009, 2010b) in the root of rice under low phosphorus stress shows that the differentially expressed genes also participate in different functional category i.e., metabolism, secondary metabolism signaling, stress DNA synthesis, transcription, transport, lipids metabolism, the **P** transport. Similarly, in our study, a number of genes are found to be participating in above said functions, which infers that similar reaction mechanism might be occurring under the **P** deficiency whether they are soil-grown or *in vitro*. It can also be inferred that the less number of gene expressions occurring in soil-grown sample in comparison to *in vitro* grown sample, may be due to the relatively uncontrolled growth conditions in soil grown study in comparison to the *in vitro* grown study.

## Conclusions

The study reports transcriptome analysis using Affymetrix®GeneChip®Wheat Genome Array to illustrate the changes in gene expression profiles in soil grown nutrients deficient roots of wheat. Microarray data analysis facilitated the discovery of a large number genes expressed in wheat roots grown with limited **K, N, P, Mg**, and **S** nutrient controlled plots. The Hierarchical clustering and PCA were carried out to ensure the overall quality of the normalized samples (Figure 1A). Further, the differentially expressed 398 NRGs were identified through statistical analysis. The NRGs responsible for more than one ND were identified and the functional mechanism for each of such candidate was also explored. The SEA provides a GO based classification of NRGs and hence classified them accordingly into respective biological processes, cellular components, and molecular functions (Figure 4). MapMan based analysis illustrates the specific role of each gene in different metabolic pathways. Different metabolic pathways (Table 1) occurring in wheat root got affected due to multiple NDs which deciphers the role of an individual gene in multiple pathways. In addition, a meta-analysis of NRGs was carried out to provide a concrete understanding of gene expression potential with respect to selected root specific 109 samples (Table S5), generated under biotic/abiotic stress. Further, the study describes the major role of characterized/uncharacterized protein/enzyme under nutrients deficiency and abiotic stress condition. Finally, the variation in genes expression level *in vitro* and soil-grown environment was discussed for the entire set of candidate genes. Overall, the study illustrates set of known genes along with a set of novel candidate genes expressed under nutrients deficiency, which opens a new arena for plant biotechnologist to study and identify their specific functions.

## Ethics statement

This research does not carry out any experiment on human and animals. All data used in this *in silico* work were collected from the open sources. Hence, authors declare that there is no compliance with ethical standards.

## Author contributions

Study was conceived and designed by: SG and PV; Work was performed and analyzed by SG, BY, and PV; Manuscript prepared by: SG, UR, SF, and PV.

### Conflict of interest statement

The authors declare that the research was conducted in the absence of any commercial or financial relationships that could be construed as a potential conflict of interest.

## Supplementary material

Figure S1 Boxplots of gene expression levels in the wheat in response to different nutrients deficiency. The left y-axis shows the normalized intensity values and x-axis represents the three replicates (R1, R2, and R3) for Control, Potassium (K), Magnesium (Mg), Nitrogen (N), Phosphorus (P), and Sulfur (S).

Figure S2 Hierarchical clustering of all array samples data. The array data were examined by the hierarchical clustering method with two views of the clusters: group of treatment view and gene tree view. The group of tree consists of 6 experimental groups i.e. Control, Potassium deficiency (ND_K), Magnesium deficiency (ND_Mg), Phosphorus deficiency (ND_P), Nitrogen deficiency (ND_N), and Sulfur deficiency (ND_S).

Figure S3 Percentage expression potential of each differentially expressed probes in 109 roots specific samples of wheat available in different expression databases.

Figure S4 Tissue specific potential gene expression of all probe sets against selected root samples. The expression potential of the genes in a tissue sample of wheat is measured in term of percentages value, which is shown in color key.

Figure S5 Heat map diagram for all probe sets while comparing with root specific samples. The green and red colors specify down-regulation (log2 [2.5]) and up-regulation (log2 [-2.5]) respectively as shown in the color bar. Similarity search was done using subsets of individual stress and nutrient deficiency conditions such as heat, cold, drought/dehydration, salt, submergence, and shift from aerobic to anaerobic germination cold and drought. The expression data were obtained using Genevestigator (Zimmermann et al., 2008).

Table S1 List of NRGs expreesed in wheat root under different nutrients deficiency conditions and their fold change value and other annotated explanation.

Table S2 Probes with annoteted Gene ID and description, playing role in atleast two nutrients deficiency conditions. Blank box represent that particular gene dose not expressed in respective deficiency.

Table S3 Key NRGs associated with different metabolic pathways under all nutrient deficiencies.

Table S4 Identified hypergeometric *P*-values of genes associated with different metabolic pathways under all Nutriant dificiencies. The P value is calculated to identify the probability of perticular gene/gene group is over-represented within a functional bin at a rate higher than chance expectation. The gene/gene group has P value <0.05 was considered for futher analysis.

Table S5 List of root specific samples used in Meta-analysis during Single Experiment Analysis and Compendium Wide Analysis of Genevestigator tool.

## Abbreviations

K: Potassium
Mg: Magnesium
N: Nitrogen
P: Phosphorus
S: Sulfate
ND: Nutrient Deficiency
NRGs: Nutrient Deficiency Response Genes
RMA: Robust Multi-Array Analysis
ATP: Adenosine Triphosphate
QC: Quality Check
QA: Quantitative Analysis
PCA: Principal Component Analysis
GO: Gene Ontology
SEA: Singular Enrichment Analysis
ROS: Reactive oxygen species
LEA: Late Embryogenesis Abundant Protein
XTH: Xyloglucan endotransglucosylase/hydrolases.
