# Supplementary figures and images for: Transcriptomic Analysis of Soil Grown T. aestivum cv. Root to Reveal the Changes in Expression of Genes in Response to Multiple Nutrients Deficiency
Source: Front Plant Sci. 2017 Jun 22;8:1025. doi: 10.3389/fpls.2017.01025 (PMC5479913; doi:10.3389/fpls.2017.01025)

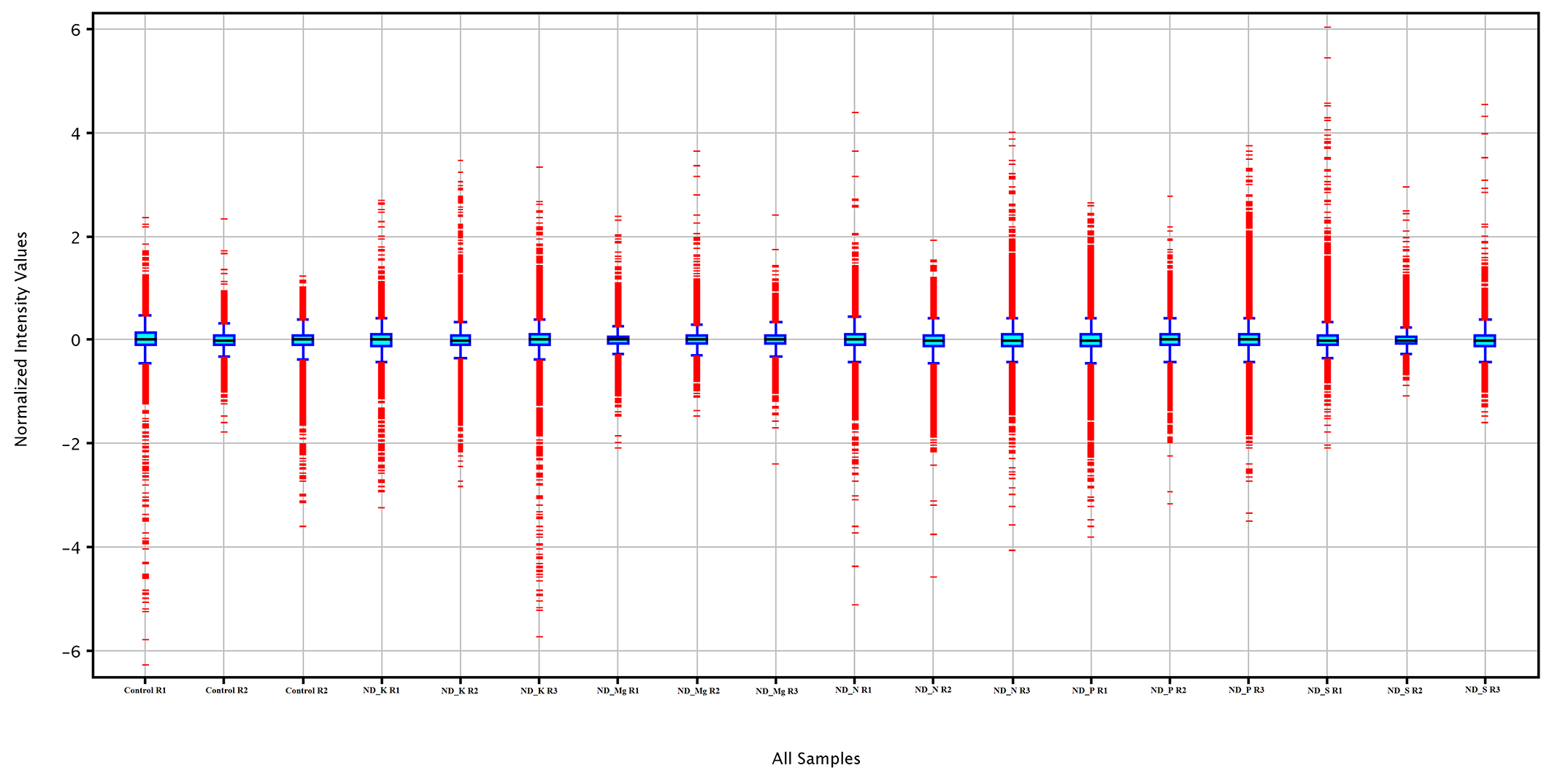

Supplement: Figure S1 — Boxplots of gene expression levels in the wheat in response to different nutrients deficiency. The left y-axis shows the normalized intensity values and x-axis represents the three replicates (R1, R2, and R3) for Control, Potassium (K), Magnesium (Mg), Nitrogen (N), Phosphorus (P), and Sulfur (S). [file Image1.TIF]

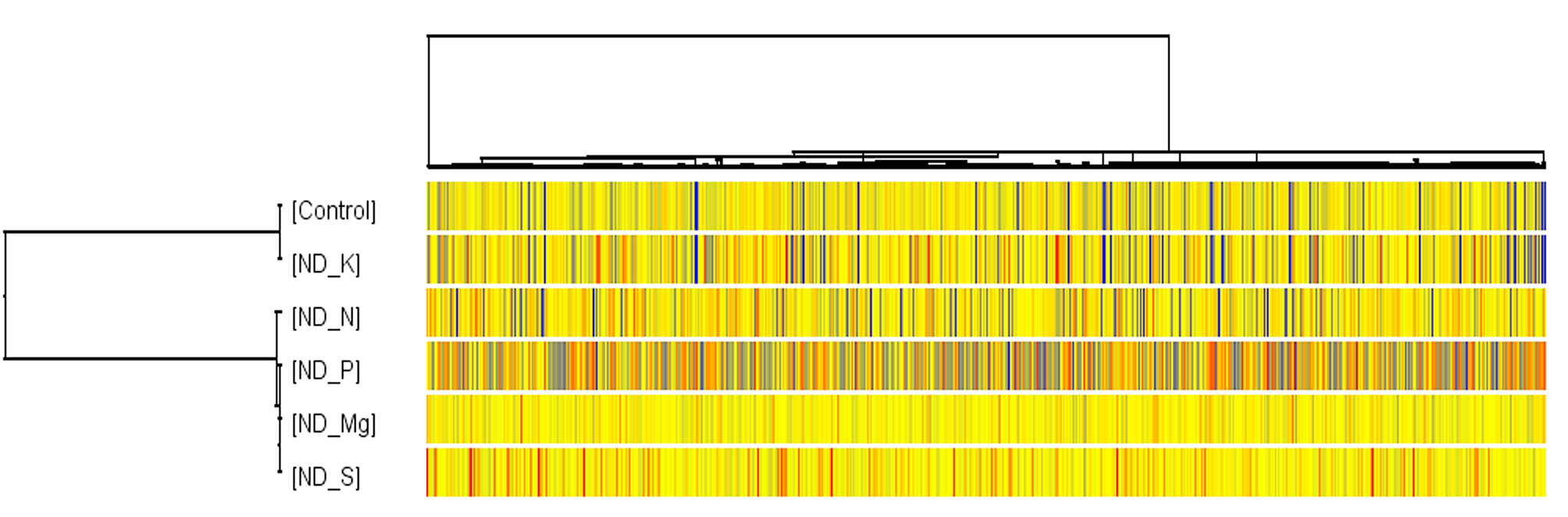

Supplement: Figure S2 — Hierarchical clustering of all array samples data. The array data were examined by the hierarchical clustering method with two views of the clusters: group of treatment view and gene tree view. The group of tree consists of 6 experimental groups i.e. Control, Potassium deficiency (ND_K), Magnesium deficiency (ND_Mg), Phosphorus deficiency (ND_P), Nitrogen deficiency (ND_N), and Sulfur deficiency (ND_S). [file Image2.TIF]

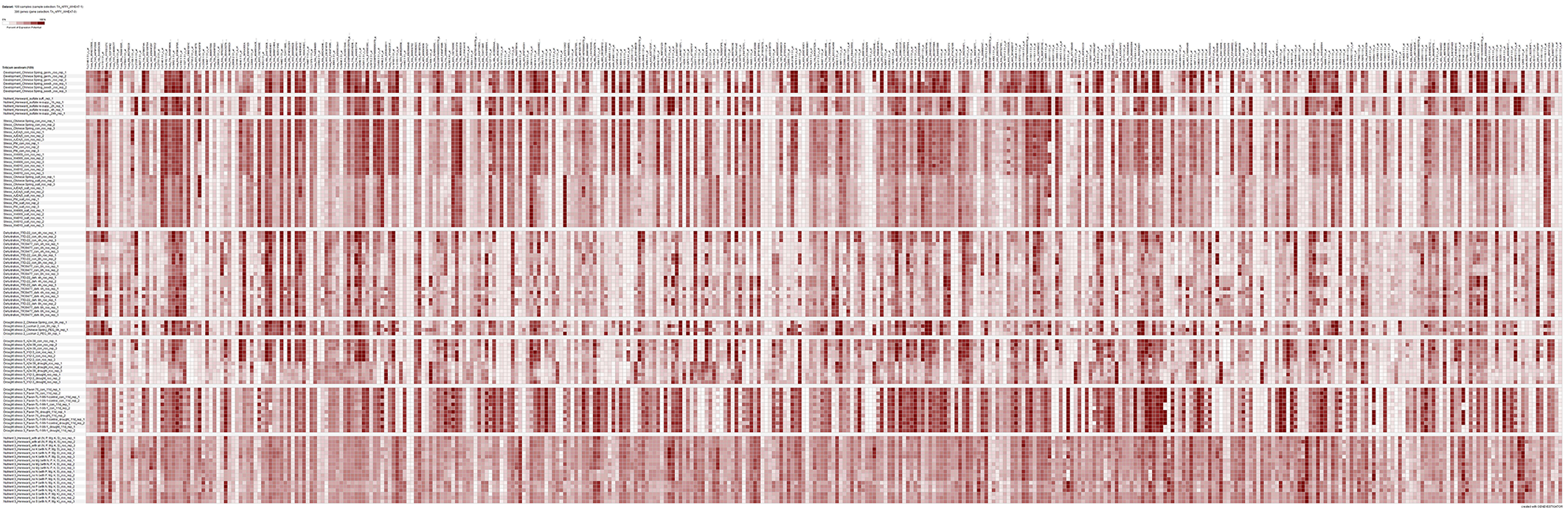

Supplement: Figure S3 — Percentage expression potential of each differentially expressed probes in 109 roots specific samples of wheat available in different expression databases. [file Image3.TIF]

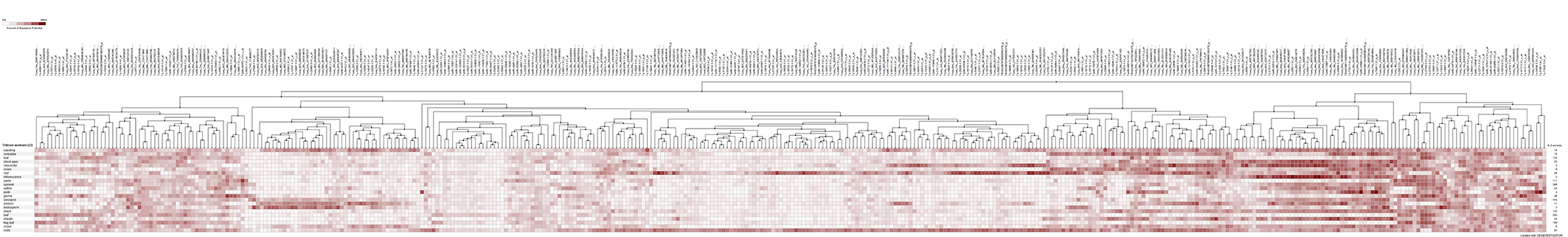

Supplement: Figure S4 — Tissue specific potential gene expression of all probe sets against selected root samples. The expression potential of the genes in a tissue sample of wheat is measured in term of percentages value, which is shown in color key. [file Image4.TIF]

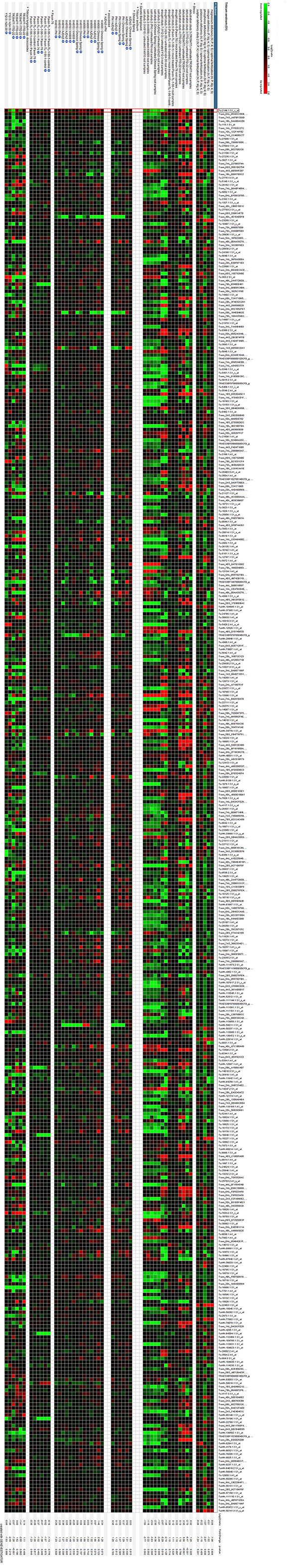

Supplement: Figure S5 — Heat map diagram for all probe sets while comparing with root specific samples. The green and red colors specify down-regulation (log2 [2.5]) and up-regulation (log2 [-2.5]) respectively as shown in the color bar. Similarity search was done using subsets of individual stress and nutrient deficiency conditions such as heat, cold, drought/dehydration, salt, submergence, and shift from aerobic to anaerobic germination cold and drought. The expression data were obtained using Genevestigator (Zimmermann et al., 2008). [file Image5.TIF]
